# Critical illness polyneuropathy (CIP) in neurological early rehabilitation: clinical and neurophysiological features

**DOI:** 10.1186/s12883-016-0775-0

**Published:** 2016-12-15

**Authors:** Simone B. Schmidt, Jens D. Rollnik

**Affiliations:** Institute for Neurorehabilitation Research (InFo), Hannover Medical School, BDH-Clinic Hessisch Oldendorf, Greitstr. 18-28, Hessisch Oldendorf, 31840 Germany

**Keywords:** Critical illness polyneuropathy, CIP, Early rehabilitation, Diagnosis, Pathophysiology, Outcome

## Abstract

**Background:**

Critical illness polyneuropathy (CIP) is a complex disease affecting 30–70% of critically ill patients.

**Methods:**

Clinical (Barthel index, length of stay (LOS), morbidity, duration of mechanical ventilation, routine lab results) and neurophysiological (neurography) data of 191 patients admitted to neurological early rehabilitation and diagnosed with CIP have been analyzed retrospectively.

**Results:**

CIP diagnosis was correct in 159 cases (83%). In this study, systemic inflammation, sepsis, systemic inflammatory response syndrome (SIRS), multiple organic failure (MOF), chronic renal failure, liver dysfunction, mechanical ventilation, diabetes, dyslipidemia and impaired ion homeostasis (hypocalcaemia, hypokalemia) were associated with CIP. Neurography, in particular of the peroneal, sural, tibial and median nerves, helped to identify CIP patients. Compound muscle action potential amplitude (*r* = −0.324, *p* < 0.05), as well as sensory (*r* = −0.389, *p* < 0.05) and motor conduction velocity (*r* = −0.347, *p* < 0.05) of the median nerve correlated with LOS in neurological early rehabilitation but not with outcome measures.

**Conclusions:**

In most cases, diagnosis of CIP among neurological early rehabilitation patients seems to be correct. Neurography may help to verify the diagnosis and to learn more about CIP pathophysiology, but it does not allow outcome prediction. Further studies on CIP are strongly encouraged.

## Background

Among critically ill neurological or neurosurgical patients entering early rehabilitation, critical illness polyneuropathy (CIP) and/or myopathy (CIM) are frequent disorders. It has been shown that CIP affects 30–70% of critical care patients [1]. CIP is regarded as a predominantly distal, motor and sensory axonal polyneuropathy [1] and may contribute to a failure of weaning from mechanical ventilation, higher mortality and prolonged length of stay (LOS) in hospital and rehabilitation [2–4]. CIP prevalence in early rehabilitation is higher than in acute-care facilities because critical care patients after failure of weaning accumulate in rehabilitation centers [5, 6].

Pathophysiology of CIP is complex and involves impaired microcirculation (sepsis), increased expression of E-selectin, cytokine secretion, increased cell permeability, mitochondrial dysfunction with reduced adenosine triphosphate synthesis (cytopathic hypoxia), damage through neurotoxic factors (reactive oxygen species, nitric oxide) and hyperglycemia [2, 7].

In addition, some risk factors have been identified, such as systemic inflammatory response syndrome (SIRS), sepsis, multiple organ failure (MOF), age, gender, mechanical ventilation, morbidity, renal failure, hypotension, hyperosmolarity, parenteral nutrition, low serum albumin, immobilization, medication and hypoxia [2, 7–9].

Until now, there are no compulsory diagnostic criteria, but it has been suggested to perform neurophysiological measurements to confirm diagnosis of CIP, in particular of the peroneal and sural nerve [10–12]. An amplitude reduction of the compound muscle action potential (CMAP) of the peroneal nerve was found to be most sensitive and specific [10].

The present study focused on clinical features and outcome of CIP patients admitted to neurological early rehabilitation.

## Methods

Patients, who have been admitted to early rehabilitation between 2004 and 2014, were screened for the main or co-diagnosis of CIP (ICD-10: G62.80). Polyneuropathies induced by alcohol, drugs or exposure to other toxic substances (G62.1; G62.0; G62.2) were not analyzed. Selected CIP cases have been carefully reviewed. Patients with prior neuromuscular disorders such as myasthenia gravis, amyotrophic lateral sclerosis or multiple sclerosis were not included. Initial lab results as well as patient clinical complexity level (PCCL), duration of mechanical ventilation, Barthel index (BI), LOS, co-diagnoses and colonization with multi-drug resistant germs (methicillin resistant staphylococcus aureus and/or extended spectrum beta-lactamase producing germs) were included in the analysis. Colonization with these bacteria is of importance because it has been demonstrated that it deteriorates outcome from neurological early rehabilitation [13, 14]. In addition, medical reports of the transferring hospital have been reviewed with respect to duration of ventilation, MOF (at least 2 or more dysfunctional organs) and sepsis. Furthermore, presence of spinal lesions were verified by existing CT or MRT images (if available) to exclude patients with palsies caused by spinal injuries. Available neurography data of the tibial, peroneal, median and sural nerve have been analyzed. For the nerve conduction studies, surface electrodes and a Nicolet Viking Select apparatus (Natus Medical, Middleton, WI, USA) were used. Motor and sensory nerve conduction velocity (CV) [m/s], distal motor latency (DML) [ms], compound muscle action potential (CMAP) amplitudes [mV] and sensory nerve action potential (SNAP) amplitudes [μV] have been recorded.

CIP diagnosis was confirmed by means of neurography and/or clinical examination (e.g. palsies, loss of tendon reflexes, sensory involvement). The neurographical examination was performed by an experienced medical practitioner, who used the following reference levels for detecting abnormal parameters: CV >40 m/sec (for tibial and peroneal nerve) or >45 m/sec (for median nerve); CMAP >5 mV (for tibial und median nerve) or >2 mV (for peroneal nerve).

Statistics were performed with SPSS version 21. First, important features were identified by descriptive statistics. Secondly, correlation analyses using the Pearson (normal distribution) or Spearman method (not normally distributed) were done comparing neurophysiological data (CMAP, SNAP, CV) and clinical or laboratory findings. In addition, the study sample was divided into two groups: confirmed vs. non-confirmed CIP diagnosis. Differences between these groups were analyzed by t-tests (normal distribution) or Wilcoxon tests (not normally distributed). *P*-values of less than 0.05 were regarded as statistically significant.

## Results

Among 191 patients transferred to early rehabilitation diagnosed with CIP, 159 cases (83.2%) were confirmed clinically (*n* = 103) and/or by neurography (*n* = 56). In 32 cases (16.8%), CIP could not be verified by neurography (*n* = 2) or by clinical examinations (*n* = 30). Within the clinical preclusions, patients with positive Babinski signs (12 cases), slight strength reduction (7 cases), flabby tonicity (3 cases) or missing any clinical signs (8 cases) were defined as false positives.

There were 90 men (57%) and 69 women (43%) in the CIP verified group, aged between 28 and 86 years (mean 66 ± 11 years). During early rehabilitation, four CIP patients died (2.5%).

Table 1 presents clinically relevant co-diagnoses of patients with confirmed CIP. Cardiovascular diseases and colonization with multi-drug resistant bacteria were found to be most frequent.

**Table 1:** Co-diagnoses of patients with confirmed CIP

Co-diagnoses	Prevalence (%)
Cardiovascular diseases	123 (77)
Colonization with multi-drug resistant germs	69 (43)
Type 2 diabetes	54 (34)
Hypokalemia	52 (33)
Hypoosmolarity and hyponatremia	36 (23)
Chronic renal failure	28 (18)
Hypothyreosis	23 (15)
Hyperlipidemia	14 (9)
Alcohol abuse	9 (6)

Table 2 shows the lab results of patients with verified CIP diagnosis. C-reactive protein (CRP) was elevated in 91.7% of cases. While 52.5% had a slight (≤50 mg/l), 27.0% showed a moderate (50–100 mg/l) and 12.2% a marked elevation (>100 mg/l). 43% had been suffering from sepsis. There was a significant correlation between CRP and CMAP of the peroneal nerve (Table 3, *r* = −0.328; *p* < 0.05).

**Table 2:** Admission lab results of patients with confirmed CIP

Lab results	Mean	SD	Normal range	Proportion of lab results [%]	
				Above normal range	Below normal range
Sodium [mmol/l]	137.78	4.78	136–145		28% ↓
Potassium [mmol/l]	4.03	0.70	3.50–5.00		
Calcium [mmol/l]	2.16	0.33	2.15–2.80		45% ↓
Chlorid [mmol/l]	100.87	5.50	97–108		
Glutamat oxalacetat transaminase [U/l]	31.95	22.70	<50		
Glutamat pyruvat transaminase [U/l]	38.55	37.70	<50		
Gamma glutamyl transferase [U/l]	**233.17**	326.21	<55	71% ↑	
Cholinesterase [U/l]	5750.81	2416.25	4000–13000		
Total protein [g/dl]	6.70	0.82	6.6–8.3		
Albumine [g/dl]	**2.38**	0.59	3.4–5.0		
Creatinine [mg/dl]	0.91	0.47	0.8–1.4	23% ↑	31% ↓
Urea [mg/dl]	**41.16**	35.08	3.6–7.2	100% ↑	
High density lipoprotein [mg/dl]	**33.50**	13.23	35.0–60.0		63% ↓
Low density lipoprotein [mg/dl]	96.48	34.81	0.0–190.0		
Total cholesterol [mg/dl]	167.77	45.79	<200		
Triacylglycerol [mg/dl]	**184.54**	96.78	<150	62% ↑	
Lipase [U/l]	263.31	222.30	73–393		
Glucose [mg/dl]	**124.70**	42.83	70–110	50% ↑	
C-reactive protein [mg/l]	**74.75**	331.56	<6	92% ↑	
Trijodthyronin GT3 [ng/ml]	0.75	0.28	0.70–1.90		
free Trijodthyronin FT3 [pg/ml]	2.17	0.67	2.18–3.98		
free Tetrajodthyronin FT4 [ng/dl]	1.23	0.36	0.60–1.50		
Thyreotropin [mU/l]	2.40	4.21	0.34–4.82		
White blood cells [10^3/μl]	9.91	4.39	4.0–10.0		
Red blood cells [10^6/μl]	**3.61**	0.54	4.40–5.90		
Platelets [10^3/μl]	336.81	133.48	150–450		
Lymphocytes [10^3/μl]	1.79	0.93	1.5–3		
Monocytes [10^3/μl]	**0.86**	0.51	0.28–0.5		
Neutrophils [10^3/μl]	**7.14**	4.09	3.2–6.2		

Values out of the normal range are highlighted in bold. ns = not significant

**Table 3:** Correlations between neurography and lab results among patients with confirmed CIP

Lab result	Peroneal nerve sural nerve			Tibial nerve	Median nerve		
	CMAP amplitude	CV	SNAP amplitude	CMAP amplitude	CMAP amplitude	Motor CV	SNAP amplitude
CRP	*p* = 0.018 *r* = −0.328^a^	ns	ns	ns	ns	ns	ns
HDL	ns	ns	ns	*p* = 0.047 *r* = 0.372^b^	ns	ns	ns
TC	ns	ns	ns	ns	*p* = 0.011 *r* = −0.352^b^	ns	ns
Lipase	ns	ns	ns	ns	ns	*p* = 0.044 *r* = −0.285	ns
Glucose	*p* = 0.028 *r* = −0.306^a^	ns	ns	ns	ns	ns	ns
Red blood cells	ns	ns	ns	ns	*p* = 0.025 *r* = 0.310^b^	ns	ns
Calcium	*p* = 0.016 *r* = 0.331^a^	*p* = 0.032 *r* = 0.301^a^	ns	ns	ns	ns	ns
Potassium	ns	ns	ns	ns	*p* = 0.022 *r* = −0.318^b^	ns	ns
Sodium	ns	ns	*p* = 0.011 *r* = 0.360^b^	ns	ns	ns	ns
Chlorid	ns	ns	*p* = 0.01 *r* = 0.374^b^	ns	ns	ns	ns
Creatinin	ns	ns	ns	ns	ns	ns	*p* = 0.034 *r* = −0.295^a^

^a^Correlation analysis by Spearman (not normal distributed data), ^b^Correlation analysis by Pearson (normal distribution)

MOF had occurred in 14% (*n* = 21) of all cases and was associated with a CV slowing of the peroneal nerve (*p* < 0.05). Consistent with the diagnosis of previous MOF, increased urea and elevated gamma glutamyl transferase (GGT) levels were observed (Table 2). Chronic renal failure was detected in 18% (*n* = 28) of all cases. Patients with chronic renal failure were significantly older (*p* < 0.05), had a higher PCCL (*p* < 0.01) and smaller CMAP of the peroneal nerve (*p* < 0.01) than patients with normal kidney function.

There was a negative correlation between potassium and median nerve CMAP amplitudes (*r* = −0.318, *p* < 0.05). While mean potassium concentration was within normal range, sodium was decreased in about one third of cases (Table 2) and correlated significantly with sural SNAP amplitudes (*r* = 0.360, *p* < 0.05). As with sodium, calcium concentrations were frequently below normal range (in 45% of cases, Table 2). Calcium correlated significantly with peroneal nerve CMAP amplitudes (*r* = 0.331, *p* < 0.05) as well as CV (*r* = 0.301, *p* < 0.05).

In the present study, 78.6% of CIP patients had been on mechanical ventilation. Ventilation before or during early rehabilitation was associated with a significantly longer LOS (*p* < 0.05), sepsis (*p* < 0.01), SIRS (*p* < 0.01) and clinical sensory disturbances (*p* < 0.05). In addition, duration of ventilation (in hours) correlated negatively with median nerve CMAP amplitudes (*r* = −0.293, *p* < 0.05), motor (*r* = −0.538, *p* < 0.001) and sensory CV (*r* = −0.516, *p* < 0.001). Monocytes (*p* < 0.05), leucocytes (*p* < 0.01), neutrophils (*p* < 0.05), potassium (*p* < 0.01), urea (*p* < 0.01) and GGT levels (*p* < 0.05) were elevated in ventilated compared to spontaneously breathing patients.

Fasting glucose levels were increased in the study sample (Table 2) and correlated negatively with peroneal CMAP amplitudes (*r* = −0.306, *p* < 0.05, Table 3). Diabetic patients (type 1 (*n* = 2), type 2 (*n* = 52), Table 1) had reduced CMAP (*p* < 0.05) and SNAP amplitudes of the median nerve (*p* < 0.01). In addition, diabetic patients showed increased triacylglyceride (TG) levels (*p* < 0.05). In the whole study sample, mean TG level were increased (Table 2) and high density lipoproteine (HDL) levels were frequently decreased (Table 2) correlating with CMAP amplitudes of the tibial nerve (*r* = 0.372, *p* < 0.05). Total cholesterol levels (TC) showed a significant correlation with CMAP amplitudes of the median nerve (*r* = −0.352, *p* < 0.05) and levels of lipase correlated with motor CV of the median nerve (*r* = −0.285, *p* < 0.05, Table 3). Nevertheless, disturbance of lipoprotein metabolism was also associated with reduced number of leukocytes (*p* < 0.05), thrombocytes (*p* < 0.05) and neutrophils (*p* = 0.026) as well as reduced SNAP amplitudes of the median nerve (*p* < 0.05). In contrast, creatinine level (*p* < 0.01) was increased.

Outcome and prognosis of patients with confirmed CIP was not different from patients without neuropathy. In addition, Barthel index (BI) on admission, at discharge and BI changes (discharge minus admission) did not show any significant correlation with neurophysiological data.

LOS in neurological early rehabilitation correlated significantly with CMAP (*r* = −0.324, *p* < 0.05), motor (*r* = −0.347, <0.05) and sensory CV (*r* = −0.389, *p* = 0.004) of the median nerve.

## Discussion

The current study focused on clinical and nerve conduction features of critically ill patients diagnosed with CIP. While literature findings suggest that female gender might be an independent risk factor for CIP [15], there were no significant clinical or neurophysiological differences between men and women. Mortality in our study (2.5%) was in line with previous results from a literature review suggesting no increase of unadjusted mortality in critical ill patients with neuromuscular abnormalities [9]. However, other studies revealed a higher mortality between 21 and 55% among CIP and CIM patients [10–12], in particular with coincident sepsis [16, 17].

Confirmed risk factors for CIP are sepsis, SIRS and MOF [7, 11, 18–20]. CRP, a marker for SIRS [21] and sepsis [22, 23], were increased in nearly all cases of the present study, indicating systemic inflammation. Incidence of sepsis (*n* = 68; 43%) was higher than reported rates in other studies [11]. We found a significant negative correlation between CRP and CMAP amplitudes of the peroneal nerve suggesting that nerve damage might be related to inflammatory mechanisms. One reason for this correlation could be the fact that the peroneal nerve shows most sensitivity for CIP diagnosis [10] and therefore most sensitivity for inflammatory processes. MOF (*n* = 21; 14%) was associated with decreased motor CV of the peroneal nerve.

Consistent with MOF, increased uric acid, decreased creatinine and elevated gamma glutamyl-transferase (GGT) levels indicating renal and liver dysfunction were observed in the current study. Chronic renal failure were detected in 18% (*n* = 28) of all cases, which was twice as much than reported previously [11]. In addition, patients with chronic renal failure were older, exhibited higher morbidity (PCCL) and had reduced CMAP amplitudes of the peroneal nerve in our study. In this case, reduced CMAP of peroneal nerve could be caused by toxic effects of uremic toxins upon the nerve axon membrane and not necessarily by CIP. However, it has been hypothesized that renal damage raises level of endoneurial potassium (even though serum potassium levels stay in normal range), affecting slow potassium channels and leading to membrane depolarization and pathogenesis of CIP [1]. In line with this finding, the present study demonstrated significant negative correlations between potassium level and median nerve CMAP amplitudes (*p* < 0.05). In healthy volunteers, a high potassium level was associated with lower excitability of the median nerve [24, 25]. Moreover, membrane depolarization and pathogenesis of CIP may be induced by hyperkalemia [26, 27]. While potassium level was within normal range, sodium level was increased in this study and significantly correlated with SNAP amplitudes of the sural nerve.

With respect to ion homeostasis, hyper- but in particular hypocalcemia are independent risk factors for CIP [28]. In this study, calcium levels were below normal. Furthermore, calcium levels correlated with peroneal CMAP amplitudes and motor CV. These findings suggest that hypocalcemia might play a role in the pathophysiology of CIP, probably by changes of membrane polarization [29].

It is known that mechanical ventilation is associated with CIP [9, 18]. In this study, 79% of all patients had been on mechanical ventilation before or during early rehabilitation. Ventilation was associated with a longer LOS (*p* < 0.05), which is in line with a previous study [11]. In addition, ventilated patients suffered from sepsis (*p* < 0.01), SIRS (*p* < 0.01) and sensory impairment (*p* < 0.05) more frequently. The longer the patients were on mechanical ventilation, the smaller were the recorded CMAP amplitudes and the lower were the motor and sensory CV of the median nerve. This finding supports the hypothesis that ventilation is a risk factor for the emergence of CIP [9].

Diabetes mellitus is a common cause of polyneuropathy [30, 31] and might confound findings of CIP. Fasting glucose levels were elevated in the study sample and correlated with peroneal CMAP amplitudes. Hyperglycemia as a result of reduced insulin production caused by increased glucagon synthesis (postagression metabolism) might be one explanation for the increased glucose levels. However, it should be taken into account that the postagression phase is normally observed within the fourth to seventh day after acute event and not during rehabilitation. In addition, there were reduced median nerve CMAP and SNAP among diabetic patients suggesting that the median nerve should be examined additionally to confirm CIP diagnosis in diabetic patients.

In line with previous studies, diabetic patients showed increased triacylglyceride (TG) [32, 33]. It is unclear whether dyslipidemia may be a risk factor for CIP. In the current study, high density lipoproteine (HDL) levels were below normal and correlated positively with tibial nerve CMAP amplitudes. Reduced HDL level are associated with immobility, which is associated with CIP. Therefore, a relationship between HDL and reduced CMAP of tibial nerve is maybe caused by immobility. Other studies discovered prevalence of hypercholesterolemia [19] and increased TG level [34]. In the current study, TC correlated negatively with CMAP of the median nerve. It remains unclear why the correlation was detected only in the median and not in the peroneal nerve. Maybe the median nerve is specific for hypercholesterolemia. Other studies demonstrated elevated total cholesterol levels in patients with carpal tunnel syndrome and reported that hypercholesterolemia is an independent risk factor [35, 36]. Therefore, a direct causal link between CIP and increased total cholesterol levels probably is non-existent. However, the role of lipid metabolism is a matter of controversial discussion [37].

CIP is regarded as a predominantly axonal polyneuropathy [1], but there was also evidence for a demyelinating nerve impairment (CV reduction) in our study. Confounding diagnoses like diabetes might account for this phenomenon and it is difficult to extrapolate these influences. However, given the broad spectrum of risk factors in CIP, it would be surprising to find nothing but axonal nerve damage. The results from our study suggest that primary or secondary demyelinating processes may occur in CIP, too.

Clinical neurophysiology, in particular evoked potentials, may be helpful to predict outcome of early rehabilitation patients [38–40]. However, neurography data of CIP patients did not correlate with any of the outcome measures. While duration of mechanical ventilation had an influence on the extent of axonal and demyelinating nerve damage, neurography did not predict functional independence of CIP patients.

Most frequently, neurography of peroneal and sural nerves have been analyzed in CIP [10–12]. Our data confirmed the importance and sensitivity of peroneal nerve measurements [10]. However, results from our study suggest that examination of tibial and, in particular, median nerve conduction may be of importance wheninvestigating CIP pathophysiology, too.

### Limitations

The study has some limitations. First, since the CIP diagnosis was confirmed mainly by clinical examination, the number of false positive diagnoses could be underestimated. However, in clinical settings, the diagnosis of CIP is usually confirmed through clinical examinations performed by experienced physicians. In addition, CIP and CIM often occur together and cases with a combination of CIP/CIM pathogenesis cannot be excluded. Although electromyography and nerve conduction studies provide a bedside method to confirm the diagnosis, routine electrophysiological examination cannot discriminate between CIP and CIM in critically ill, sedated, uncooperative, or extremely weak patients. A differentiation of CIP and CIM is only possible in adequately awake and cooperative patients, which is often in contrast to the state of consciousness of our patients. Therefore, the main diagnosis is made by clinical examination. However, the clinical relevance of differentiating between neuropathy and myopathy is still under debate, because the outcome prognosis is almost the same. A strict separation of CIP and CIM is only possible via muscle biopsy, which is rarely performed in daily clinical practice. Due to the study design (retrospective analysis of routinely collected data) muscle biopsies were performed only in some cases. Second, no temperature recording was performed during neurography measurement and reference values were not age-adjusted, which could have slight effects on the NC. Third, only patients with CIP main or co-diagnosis were investigated in the study. A comparison with age- and sex-matched patients with similar Barthel-Index, co-diagnosis and central lesions, e.g. the main ICD-10 diagnosis I63, would be an important benefit for the quality of the study.

## Conclusions

CIP is a complex disease with a broad spectrum of risk factors, but pathophysiology is still unclear. The current study supports the hypothesis that sepsis, SIRS, MOF, chronic renal failure, liver dysfunction, mechanical ventilation, diabetes, dyslipidemia, systemic inflammation and impaired ion homeostasis (hypocalcaemia, hypokalemia) are associated with CIP. Neurophysiological examinations of peroneal, sural, tibial and median nerves may help to study CIP pathophysiology and to confirm diagnosis, but they do not predict outcome.

## Abbreviations

BI: Barthel index
CIM: Critical illness myopathy
CIP: Critical illness polyneuropathy
CMAP: Compound muscle action potential
CRP: C-reactive protein
CV: Conduction velocity
DML: Distal motor latency
GGT: Gamma glutamyl-transferase
HDL: High density lipoprotein
LDL: Low density lipoprotein
LOS: Length of stay
MOF: Multi organ failure
PCCL: Patient clinical complexity level
SIRS: Systemic inflammatory response syndrome
SNAP: Sensory nerve action potential
TC: Total cholesterol
TG: Triacylglyceride
